# Cost-utility analysis of empagliflozin compared with dapagliflozin in patients with type 2 diabetes in China

**DOI:** 10.3389/fpubh.2025.1566101

**Published:** 2025-07-07

**Authors:** Huiyuan Zhang, Jiaojiao Chen, Quan Zhao, Bei Zhang

**Affiliations:** Department of Pharmacy, Yantai Yuhuangding Hospital, Yantai, Shandong, China

**Keywords:** cost-utility analysis, type 2 diabetes mellitus, empagliflozin, dapagliflozin, China

## Abstract

**Background:**

Empagliflozin, a sodium-glucose cotransporter 2 inhibitor, performs a reduction in the all-cause mortality and cardiovascular mortality in type 2 diabetes mellitus (T2DM) patients compared to dapagliflozin, which has been included in the national volume-based procurement in China. The objective of this study is to evaluate the long-term cost-utility of the addition of empagliflozin (10 mg or 25 mg) versus dapagliflozin (10 mg) in T2DM patients with insufficient control by metformin monotherapy from the perspective of Chinese health care payers.

**Methods:**

The IQVIA CORE diabetes model was used for cost-utility analysis to compare the long-term economics of empagliflozin (10 or 25 mg) versus dapagliflozin (10 mg) respectively. In the two independent analyses, the discount rate was 5% per year, and the utility value was derived from the published literatures. The baseline demographic and biochemical data, as well as treatment efficacy data were obtained from the EMPA-REG MET clinical trial and network meta-analysis, respectively.

**Results:**

Compared with dapagliflozin 10 mg, empagliflozin 10 mg and empagliflozin 25 mg improved the life expectancy by 0.011 and 0.02 years, and improved the quality adjusted life years (QALYs) by 0.011 and 0.02 years, respectively. The total cost of empagliflozin group (10 mg) was 279 Chinese Yuan lower than that of the dapagliflozin group (10 mg), making it an absolutely economical choice. The total cost of empagliflozin (25 mg) was expected to be 1,601 Chinese Yuan higher than dapagliflozin, with an incremental cost-utility ratio (ICUR) of 80,052 Chinese Yuan per QALY, below the set ‌willingness to pay (WTP) threshold of 85,698 Chinese Yuan per QALY.

**Conclusion:**

For T2DM patients with insufficient control by metformin monotherapy, the addition of empagliflozin 10 mg showed better efficacy and lower cost compared to dapagliflozin 10 mg, making it an absolutely economical choice. Based on the set WTP threshold, empagliflozin 25 mg was also a more cost-effective treatment option than dapagliflozin from the perspective of Chinese healthcare payers.

## 1 Introduction

Diabetes mellitus is a chronic metabolic illness with rising incidence and prevalence worldwide, which has led to considerable negative impact on the finances of countries and the health of the people. It is estimated by the International Diabetes Federation (IDF) that there are approximately 536.6 million people with diabetes around the world, in which, China has the highest number of diabetics. According to the statistics of IDF, in 2021, about 140.9 million people in China aged 20–79 suffering from diabetes, and the number of deaths related to diabetes is estimated to exceed 1.4 million each year. Diabetes mellitus and its complications not only seriously impair the health of patients, but also cause heavy economic burden to the families of patients and the whole society. It is estimated that $966 billion is spent directly on diabetes globally in 2021, with the highest projected healthcare spending in the United States, China and Brazil at $379.5 billion, $165.3 billion and $42.9 billion, respectively (1).

According to the World Health Organization (1999) classification system of diabetes etiology, diabetes is classified into four types based on etiological evidence: type 1 diabetes mellitus, type 2 diabetes mellitus (T2DM), special type diabetes and gestational diabetes (2). Among all patients with diabetes, approximately 90% belong to T2DM (3). The significant pathophysiological features of T2DM are the decrease in the ability of insulin to regulate glucose metabolism (insulin resistance) and the decreased insulin secretion (relative decrease) accompanied by functional deficiency of pancreatic beta cells (4). The progressive resistance to insulin in T2DM patients could lead to pathological activation of the immune system, causing progressive impairment to microvascular and macrovascular tissue function, thus to increase the risk of cardiovascular events, kidney complications and mortality in patients (5, 6).

According to the prevention and treatment guidelines for type 2 diabetes of China in 2020, the strategies for controlling hyperglycemia are comprehensive, including lifestyle management, blood glucose monitoring, diabetes education, and the use of hypoglycemic drugs (7, 8). Metformin is the first line drug and the basic drug in drug combinations to control hyperglycemia in T2DM patients, which is recommended to be kept in the treatment regimen of diabetes without contraindications (9). If it is difficult to achieve the standard blood glucose level based on the use of metformin alone, it is recommended to initiate the dual therapy. Sulfonylureas, glinides, alpha-glucosidase inhibitors, thiazolidinediones, dipeptidyl peptidase-4 inhibitors, sodium-glucose cotransporter 2 inhibitors (SGLT-2i), glucagon-like peptide-1 receptor agonists, and insulin are the main combination drugs, which can be selected based on the hypoglycemia, weight, economic conditions of patients and the drug accessibility.

SGLT-2i is a new type of oral hypoglycemic drug that has received high attention in recent years. It can inhibit the reabsorption of glucose by the kidneys, reduce the renal glucose threshold, and promote the excretion of urine glucose (10). At present, the SGLT-2i inhibitors on the market in China include dapagliflozin, empagliflozin, canagliflozin, and ertugliflozin, which can be used alone or in combination with other hypoglycemic drugs to treat the T2DM of adult. SGLT-2i has shown cardiovascular and renal benefits in a series of large studies of cardiovascular and renal outcomes, including the cardiovascular outcome trial of empagliflozin (EMPA-REG OUTCOME) (11), the canagliflozin cardiovascular assessment study (CANVAS) (12), the effect of dapagliflozin on cardiovascular events (DECLARE-TIMI 58) (13), the evaluation of ertugliflozin efficacy and safety cardiovascular outcomes trial (VERTIS CV) (14), and canagliflozin and renal events in diabetes with established nephropathy clinical evaluation (CRENDENCE) (15). In patients with T2DM, SGLT-2i combined with metformin has been shown to be associated with the reduction of cardiovascular disease (CVD) events, particularly in those patients who already have atherosclerotic CVD. Some international guidelines recommend SGLT-2i as the first-line treatment for T2DM. For example, the American Diabetes Association standards of care in diabetes in 2025 recommended SGLT-2i as the first-line treatment for patients who have been identified or are at high risk for cardiovascular disease, regardless of the blood glucose level and the use of metformin (16). In addition, the current 2023 European Society of Cardiology guidelines for the management of cardiovascular disease in patients with diabetes recommend SGLT-2i as first-line treatment for T2DM patients with or at high risk for cardiovascular disease, whether they are untreated or treated with metformin alone (17).

Among all SGLT-2i, dapagliflozin is the first drug approved for marketing, which has a high market share in China and has been included in the national essential medicines list. However, empagliflozin has shown to be superior to canagliflozin and dapagliflozin in reducing cardiovascular mortality and all-cause mortality in patients with T2DM (18, 19). Since 2018, China has implemented a centralized drug procurement policy named the national volume-based procurement (NVBP), attempting to reduce drug prices and alleviate the treatment burden for patients through competitive bidding, bulk procurement, and reducing transaction costs. So far, 10 batches of NVBP have been carried out; involving some oral hypoglycemic drugs such as empagliflozin and metformin, but dapagliflozin has not yet been included. It is crucial to take clinical and economic evidences into consideration to make treatment drug choices for T2DM patients, so that the healthcare providers can maximize the use of resources and optimize patient care. In the past 20 years, China has made important progress in high-quality health economic evaluation of diabetes. The clinical and economic impact of empagliflozin as a second-line treatment strategy after treatment failure with metformin has been studied in literatures. However, there is limited research evaluating the economic benefits of empagliflozin versus dapagliflozin in poorly controlled T2DM patients with metformin monotherapy. Under the implementing of NVBP, it is necessary to evaluate the economics of empagliflozin and dapagliflozin, providing valuable reference for the selection of oral hypoglycemic drug treatment plans for T2DM patients in China.

This objective of this study is to evaluate and compare the cost-utility of adding empagliflozin versus dapagliflozin to T2DM patients with insufficient control of metformin monotherapy in the Chinese healthcare environment from the perspective of healthcare system.

## 2 Methods

### 2.1 Model overview

The IQVIA CORE diabetes model (CORE model) is used for the cost-utility analysis to compare the economic efficiency of the treatment groups of empagliflozin and dapagliflozin. The CORE model is an internationally recognized health economics modeling tool for diabetes, with strong predictive capabilities and a solid foundation in model validation. It has been clarified that the IQVIA CORE diabetes model could represent the natural disease history of diabetes and offers a wider range of interactions between complications (20, 21). Compared with other models, CORE model is more suitable for the Asian population because it uses the new risk equation based on the Hong Kong Diabetes Registry study (22). This model has been widely used in diabetes economic research in multiple countries and regions, including China, which is applicable to simulating the disease progression trajectories and treatment pathways of Chinese patients (23–25). CORE model consists of 17 interdependent Markov submodels, and simulates the development of diabetes and its complications using Monte Carlo simulation technology. In this study, the simulation period is the whole life cycle of patients. The model construction takes into account the baseline characteristics, diabetes complications, current and future diabetes management, screening strategies and changes in physiological parameters over time to predict the development of complications, life expectancy, quality adjusted life years (QALYs), costs and other results inT2DM patients (26).

### 2.2 Model inputs

#### 2.2.1 Baseline cohort characteristics

A hypothetical model cohort was defined based on baseline demographic data of Chinese T2DM patients with poorly controlled metformin monotherapy. The baseline demographic data and biochemical parameters of the model cohort were obtained from the EMPA-REG MET trial, which included T2DM patients with insufficient control of metformin from a multicenter cohort (Asian patients accounted for 45%), supplemented with reference data as necessary (27). The starting age of study patients was 55.7 years old, the mean duration of diabetes was 6 years, and the baseline HbA1c level was 7.9%. Detailed baseline characteristics were listed in the supporting information (Supplementary Table S1).

#### 2.2.2 Intervention and comparator

This economic evaluation compared empagliflozin and dapagliflozin as a complementary treatment for metformin. The treatment pathways for diabetes are highly individualized, and including all possible treatment regimens would significantly increase the complexity of the model structure while introduce a large number of parameters with inconsistent sources and qualities, which would reduce the stability and interpretability of model predictions. To enhance the operability and stability of the model during long-term simulation process, this study reasonably simplified the treatment pathway. The first-line treatment for T2DM patients was metformin 0.5 g po tid, the second-line treatment is metformin (0.5 g po tid) plus empagliflozin (10 mg po qd or 25 mg po qd) or dapagliflozin (10 mg po qd), and the next level treatment was set at the combination therapy of 51 units of insulin glargine per day with metformin. When the HbA1c level exceeded the defined threshold of 7.0%, the patient switched to escalation treatment. The dosage of oral hypoglycemic drugs referred to the drug instructions and treatment guidelines; while the daily dose of insulin as a supplement of metformin was referred to a 26 week randomized trial (28). This treatment regimen aligned with the trends in Chinese clinical practice and existing pharmacoeconomic studies to ensure the rationality, feasibility and clinical representativeness of this research (23, 29, 30).

#### 2.2.3 Clinical and treatment efficacy inputs

According to the setting requirements of the CORE model, the required treatment outcomes incorporated in the analysis including changes in HbA1c from baseline, incidence of severe hypoglycemic events, and the incidence of nonsevere hypoglycemic events. Detailed treatment-associated adverse reactions such as urinary tract infections and ketoacidosis were not included. Due to the lack of randomized controlled trial data for empagliflozin and dapagliflozin, the treatment efficacy data used in the analysis was derived from a network meta-analysis (31). The therapeutic effect and incidence of adverse events of insulin glargine were based on data from published literatures. The clinical input parameters of each treatment arm were listed in Supplementary Table S2.

### 2.3 Utility inputs

The health status utility value and event disutility value of type 2 diabetes and its complications were derived from published studies, as shown in Supplementary Table S3 (23). For acute events, the final utility value of the patient was obtained through subtracting the disutility value from the basic health status utility value. If a patient suffered from multiple events simultaneously (such as myocardial infarction and stroke), the model followed the minimum approach. For utility values that cannot be obtained from literatures, the default values in the CORE model were used.

### 2.4 Cost inputs

This cost-utility analysis was conducted from the perspective of the Chinese healthcare system; therefore, only direct medical costs were considered in the analysis, including drug purchase costs, management costs of diabetes-related complications, routine management costs of patients and adverse event handling costs. The cost of empagliflozin, dapagliflozin, metformin, and insulin were calculated by multiplying the original drug price published in the local drug procurement platform by the annual dose, as shown in Table 1. Among them, the cost of empagliflozin with the specification of 25 mg of was calculated based on the price of empagliflozin with the specification of 10 mg. The management costs of diabetes and handling costs of related complications were listed in Table 2, mainly based on the published literatures (23, 32, 33). All expenses were converted into 2022 costs using the Chinese Consumer Price Index, and the unit of data was Chinese yuan (CNY).

**Table 1:** Treatment costs of different medications.

Medicine	Strength	Daily dose	Daily cost (CNY)	Annual drug costs (CNY)
Empagliflozin	10 mg	10 mg	4.24	1,548.66
Empagliflozin^†^	25 mg	25 mg	10.60^†^	3,871.65
Dapagliflozin	10 mg	10 mg	4.36	1,592.49
Metformin	0.5 g	1.5 g	2.87	1,049.18
Insulin glargine	300 IU	51 IU	11.73	4,284.38
Metformin + empagliflozin	0.5 g + 10 mg	1.5 g + 10 mg	7.11	2,597.84
metformin + Empagliflozin^†^	0.5 g + 25 mg	1.5 g + 25 mg	13.47	4,920.83
metformin + dapagliflozin	0.5 g + 10 mg	1.5 g + 10 mg	7.23	2,641.67
Metformin + insulin glargine	0.5 g + 300 IU	1.5 g + 51 IU	14.60	5,333.56

^†^The cost of empagliflozin with the specification of 25 mg was calculated based on the price of empagliflozin with the specification of 10 mg.

**Table 2:** Management costs, diabetes related complication costs, and adverse event costs.

Variable	Value (2020 CNY)	Value (2022 CNY)	References
Management costs			
Annual statins treatment	1,069	1,080	Simvastatin daily costs × 365.25
Annual Aspirin	219	221	Aspirin daily costs × 365.25
Annual ACEI	47	48	Ramipril daily costs × 365.25
Annual screening microalbuminuria	40	40	(23)
Annual screening macroalbuminuria	15	15	(23)
Annual eye screening	60	61	(23)
Foot screening program	1,464	1,479	(32)
Annual non-standard ulcer treatment	0	0	(32)
Direct costs of CVD complications			
Myocardial infarction 1st year	32,517	32,843	(23)
Myocardial infarction 2nd + years	10,839	10,948	(23)
Angina 1st year	41,245	41,658	(23)
Angina 2nd + years	7,363	7,437	(23)
Congestive heart failure 1st year	9,756	9,854	(23)
Congestive heart failure 2nd + years	6,503	6,568	(23)
Stroke 1st year	19,165	19,357	(23)
Stroke 2nd + years	8,630	8,717	(23)
Stroke death within 30 days	14,852	15,001	(23)
Peripheral vascular disease 1st year	25,390	25,645	(23)
Peripheral vascular disease 2nd + years	9,869	9,968	(23)
Direct costs of renal complications			
Hemodialysis 1st year	85,285	86,140	(23)
Hemodialysis 2 + years	72,001	72,723	(23)
Peritoneal dialysis 1st year	59,168	59,761	(23)
Peritoneal dialysis 2 + years	48,259	48,743	(23)
Renal transplant costs 1st year	256,204	258,772	(23)
Renal transplant 2 + years	67,755	68,434	(23)
Direct costs of acute events			
Non-severe hypoglycaemic event	182	184	(23)
Severe hypoglycemic event 1 (requiring non-medical assistance)	182	184	(23)
Severe hypoglycemic event 2 (requiring medical assistance)	3,869	3,908	(23)
Urinary tract infection + genital infection	9	9	(23)
Keto event	13,144	13,276	(33)
Lactic acid event	8,996	9,086	(33)
Direct costs of eye disease			
Laser treatment	2,391	2,415	(23)
Cataract operation	7,529	7,604	(23)
Following cataract operation	197	199	(23)
Blindness − year of onset	5,638	5,695	(23)
Blindness − following years	1,528	1,543	(23)
Direct costs of neuropathy/foot ulcer/amputation			
Neuropathy, 1st year	5,346	5,400	(23)
Neuropathy, 2nd + years	6,479	6,544	(23)
Amputation (lower extremity)	7,128	7,199	(23)
Amputation prosthesis	21,460	21,675	(23)
Gangrene treatment	4,753	4,801	(23)
After healed ulcer	896	905	(23)
Infected ulcer	5,346	5,400	(23)
Standard uninfected ulcer	3,262	3,295	(23)
Healed ulcer history of amputation	896	905	(23)

ACEI, angiotensin-converting enzyme inhibitor.

### 2.5 Base-case analysis

This study calculated the direct medical expenses, life years, and quality adjusted life years (QALYs) for three different treatment groups: empagliflozin (10 mg po qd), empagliflozin (25 mg po qd), and dapagliflozin (10 mg po qd) combined with metformin. The results of cost-utility analysis were evaluated by calculating the incremental cost of each QALY, namely the incremental cost-utility ratio (ICUR). Following the recommendations of the Chinese pharmacoeconomic evaluation guidelines, all costs and health outcomes were analyzed at a discount rate of 5% per year (34). According to the Chinese pharmacoeconomic evaluation guidelines that the willingness to pay threshold used for the quality adjusted life years in the incremental analysis was recommended to be 1 to 3 times the national per capita gross domestic product, this study regarded China’s per capita gross domestic product in 2022 as the willingness to pay threshold (WTP; 85,698 yuan per QALY) (34). Given that diabetes was a lifelong chronic disease, the basic case of this study was defined as a lifelong analysis. The starting age of the baseline cohort of studied patients was 55.7 years, and a 40-year time span was considered sufficient to keep most patients in a state of death. Therefore, we simulated the disease progression and health outcomes of patients over 40 years to obtain long-term economic results.

### 2.6 Sensitivity analysis

In order to evaluate the impact of changes in key parameters on the cost-utility analysis results, we conducted a series of one-way sensitivity analysis, probabilistic sensitivity analysis, and scenario analysis.

#### 2.6.1 One-way sensitivity analysis

The China guidelines for pharmacoeconomic evaluations clearly recommends that a sensitively analysis should be conducted by varying the discount rate within the range of 0–8% (34). The key parameter variations considered in the one-way sensitivity analysis (OWSA) involved the discount rate of costs (0, 3 and 8%), medication costs of oral hypoglycemic drugs (the highest or lowest bid price in national volume-based procurement), and the time span of research (10, 20, 30, and 50 years).

#### 2.6.2 Probabilistic sensitivity analysis

The probabilistic sensitivity analysis (PSA) was carried out using a Monte Carlo simulation method with 1,000 iterations.

#### 2.6.3 Scenario analysis

Additional scenarios were run to test the impact of various hypotheses on the results. The short-term cost-utility analysis results of different treatment options were evaluated by shortening the simulation time span to 4 years.

## 3 Results

### 3.1 Empagliflozin 10 mg vs. dapagliflozin 10 mg

#### 3.1.1 Base-case analysis

In the basic case analysis (Supplementary Table S4), in terms of long-term effects, empagliflozin 10 mg was associated with a discounted life expectancy advantage of 0.011 years per patient compared with dapagliflozin 10 mg (12.504 years vs. 12.493 years). In terms of quality of life, compared with the dapagliflozin treatment group, empagliflozin also showed similar benefits, with an increase of 0.011 QALYs per patient (8.974 vs. 8.963 QALYs). As shown in Table 3, for T2DM patients with insufficient control of metformin monotherapy, the addition of empagliflozin 10 mg meant an increase of CNY 13 in treatment costs compared to the combination of dapagliflozin 10 mg. However, patients receiving the treatment of empagliflozin maintained the second-line treatment for 4 years, while patients receiving dapagliflozin maintained the current treatment regimen for 3 years. After that, both groups of patients were adjusted to the combined treatment of metformin with insulin glargine. In the dapagliflozin group, patients initiated insulin glargine 1 year earlier, which resulted in a higher risk and cost for the hypoglycemia adverse events and cardiovascular disease. Therefore, the increased treatment costs in the empagliflozin group could be offset by the cost savings in other categories. Overall, the direct medical expense for patients in the empagliflozin group (10 mg) was CNY 279 lower than those in the dapagliflozin group (10 mg). In summary, for T2DM patients with poorly control of metformin monotherapy, a 40-year cycle simulation showed that the addition of empagliflozin 10 mg was an absolutely economical choice with better efficacy and lower cost compared to dapagliflozin 10 mg.

**Table 3:** Breakdown of the total costs of empagliflozin 10 mg vs. dapagliflozin 10 mg.

Cost category (CNY)	Metformin + Empagliflozin 10 mg	Metformin + Dapagliflozin 10 mg	Difference
Total cost	171,282	171,561	−279
Treatment	65,955	65,943	13
Management	22,180	22,156	24
CVD	31,385	31,481	−96
Renal	7,073	7,153	−79
Ulcer/amputation/neuropathy	37,994	38,118	−125
Eye	5,985	5,998	−13
NSHE	709	712	−3
SHE1	–	–	–
SHE2	–	–	–

NSHE, non-severe hypoglycemic event; SHE1, severe hypoglycemic event (not requiring medical assistance); SHE2, severe hypoglycemic event (requiring medical assistance).

#### 3.1.2 Sensitivity analysis

**Figure 1:**
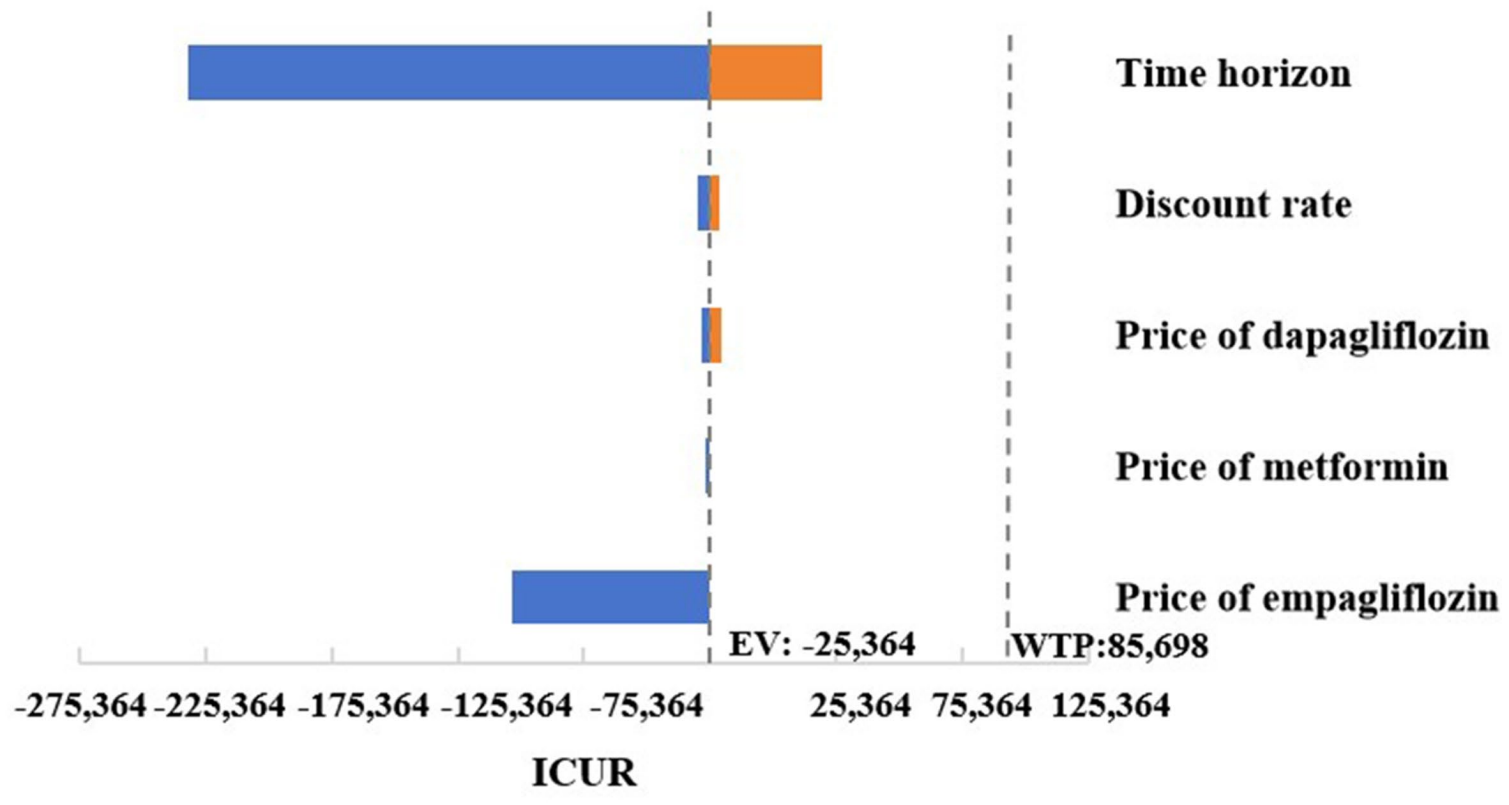
Tornado graphs of one-way sensitivity analysis for empagliflozin 10 mg vs. dapagliflozin 10 mg.

The results of a series of OWSA indicated that the changes in key parameters such as the medication costs of oral hypoglycemic drugs, different discounting rates, and simulation time spans had no significant impact on the robustness of the final results. As shown in Figure 1, in the case where the simulated research time horizon was adjusted to 30 years, compared with dapagliflozin 10 mg, empagliflozin 10 mg was associated with an increase in QALYs and costs, while the ICUR was lower than the set WTP. In other cases of the OWSA, the empagliflozin 10 mg treatment regimen always showed higher QALYs and lower costs compared to dapagliflozin, making it an absolutely economical choice.

**Figure 2:**
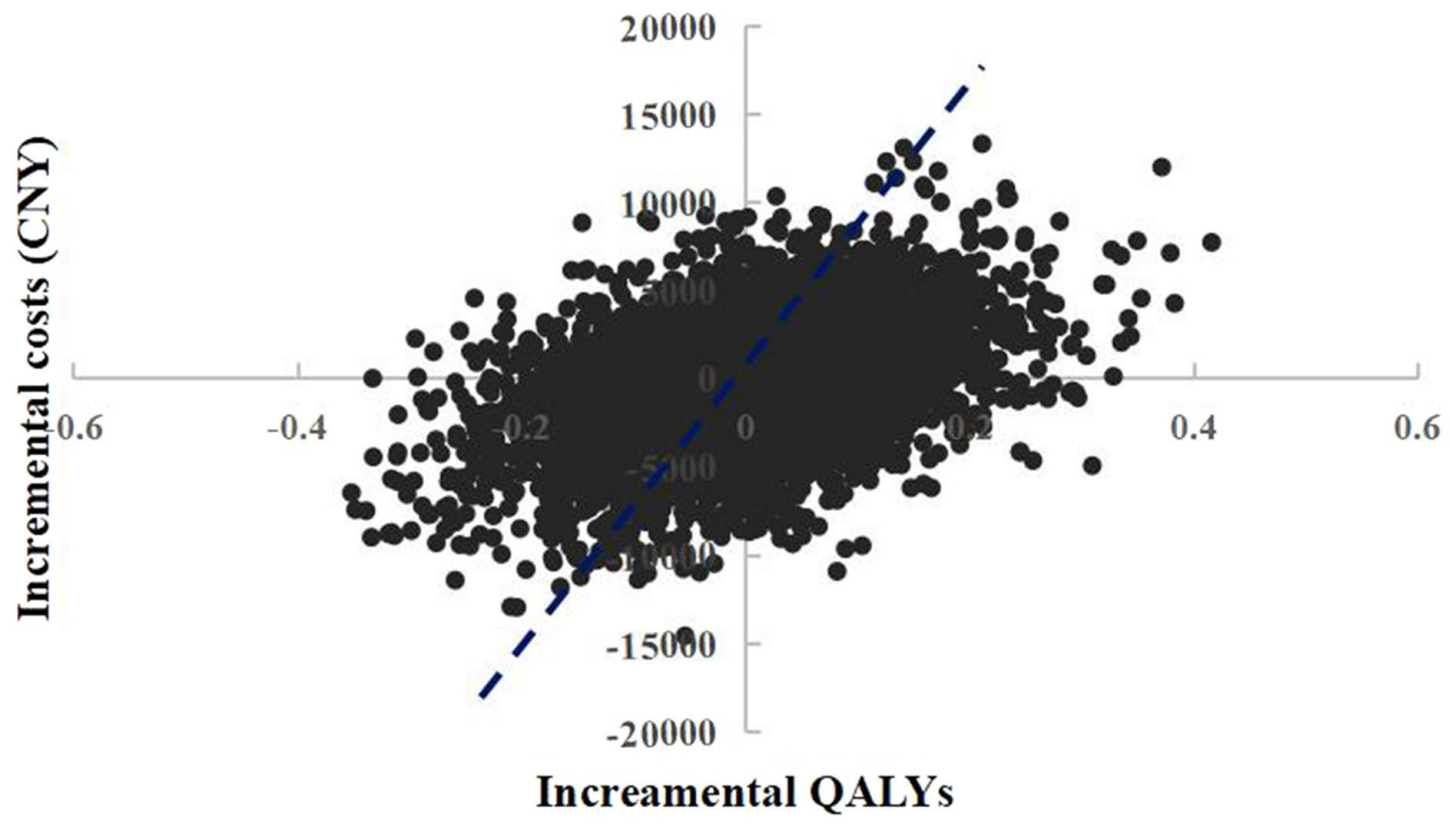
Probabilistic sensitivity analysis scatterplot (empagliflozin 10 mg vs. dapagliflozin 10 mg).

The scatter plot of change in cost and change in QALYs in PSA was shown in Figure 2. With a WTP threshold of CNY 85,698/QALY, there was a 51.3% probability that empagliflozin 10 mg was more cost-effective than dapagliflozin 10 mg.

In the short-term scenario analysis (Table 4), the treatment group supplemented with empagliflozin 10 mg also exhibited higher QALYs and lower costs, further consolidating the cost-effectiveness trend observed under baseline case.

**Table 4:** Breakdown of costs for the shorter-time scenario analyses.

Cost category (CNY)	Metformin + empagliflozin 10 mg	Metformin + dapagliflozin 10 mg	Difference
Total cost	25,478	25,665	−187
Treatment	9,106	9,260	−153
Management	6,033	6,034	−1
CVD	4,512	4,525	−12
Renal	678	673	5
Ulcer/amputation/neuropathy	4,059	4,072	−13
Eye	1,074	1,072	2
NSHE	14	29	−14
SHE1	–	–	–
SHE2	–	–	–

NSHE, non-severe hypoglycemic event; SHE1, severe hypoglycemic event (not requiring medical assistance); SHE2, severe hypoglycemic event (requiring medical assistance).

### 3.2 Empagliflozin 25 mg vs. dapagliflozin 10 mg

#### 3.2.1 Base-case analysis

Compared to 10 mg of dapagliflozin, 25 mg of empagliflozin was associated with a discounted life expectancy of 0.02 years per patient (12.513 years vs. 12.493 years). In terms of quality of life, the empagliflozin treatment group also showed similar benefits, with a benefit of 0.02 QALYs per patient (8.983 QALYs vs. 8.963 QALYs) compared to dapagliflozin. For T2DM patients with insufficient control of metformin monotherapy, the addition of empagliflozin 25 mg meant an increase of CNY 2,271 in treatment costs compared to the combination of dapagliflozin 10 mg. However, patients receiving the treatment of empagliflozin maintained the second-line treatment for 4 years, while patients receiving dapagliflozin maintained the current treatment regimen for 3 years. After that, both groups of patients were adjusted to the combined treatment of metformin with insulin glargine. In the dapagliflozin group, patients initiated insulin glargine 1 year earlier, which resulted in a higher risk and cost for the hypoglycemia adverse events and cardiovascular disease. As shown in Table 5, a portion of the increased drug costs in the empagliflozin group could be offset by savings in other expense categories. Overall, when calculating the direct medical expenses for patients, the average cost of the empagliflozin group (25 mg) was CNY 1,601 higher than that of the dapagliflozin group, with an ICUR of CNY 80,052 per QALY, which was lower than the set WTP threshold of CNY 85,698 per QALY. Therefore, for T2DM patients with insufficient control of metformin monotherapy, a 40 year cycle simulation showed that empagliflozin 25 mg treatment was a more economical choice compared to dapagliflozin 10 mg.

**Table 5:** Breakdown of the total costs of empagliflozin 25 mg vs. dapagliflozin 10 mg.

Cost category (CNY)	Metformin + empagliflozin 25 mg	Metformin + dapagliflozin 10 mg	Difference
Total cost	173,162	171,561	1,601
Treatment	68,214	65,943	2,271
Management	22,209	22,156	52
CVD	31,318	31,481	−162
Renal	6,912	7,153	−241
Ulcer/Amputation/Neuropathy	37,792	38,118	−326
Eye	6,007	5,998	9
NSHE	710	712	−2
SHE1	–	–	–
SHE2	–	–	–

NSHE, non-severe hypoglycemic event; SHE1, severe hypoglycemic event (not requiring medical assistance); SHE2, severe hypoglycemic event (requiring medical assistance).

#### 3.2.2 Sensitivity analysis

**Figure 3:**
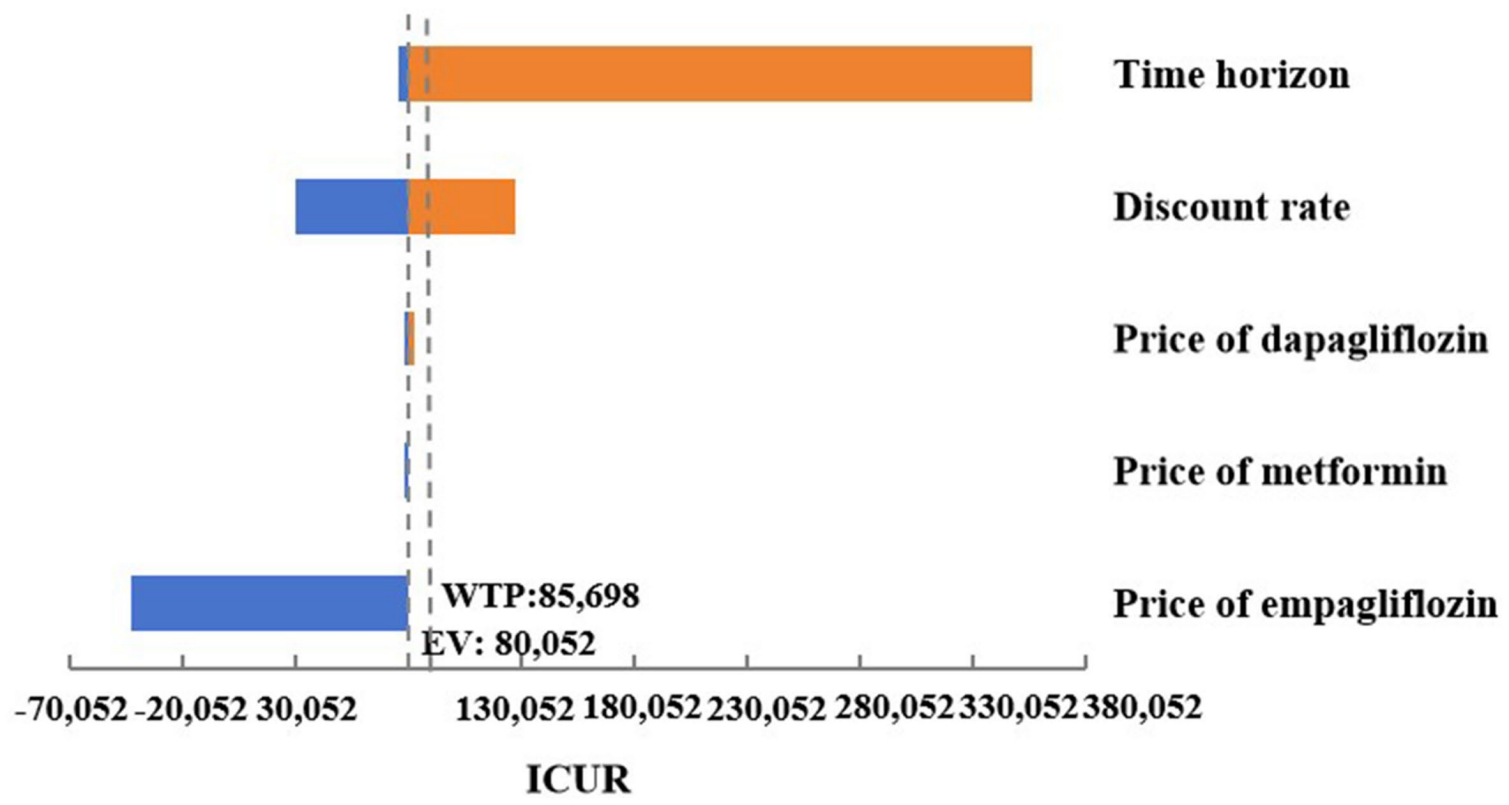
Tornado graphs of one-way sensitivity analysis for empagliflozin 25 mg vs. dapagliflozin 10 mg.

As shown in Figure 3, a series of OWSA results indicated that the medication cost of oral hypoglycemic drugs had no significant impact on the final results. However, in the cases where the simulation time span was adjusted to 10, 20, or 30 years, and the discount rate was adjusted to 8%, empagliflozin 25 mg was associated with an increase in QALYs and costs compared to dapagliflozin 10 mg, and the ICUR was higher than the set threshold of WTP. In the case where empagliflozin adopted the winning bid prices in the centralized medicine procurement, compared with dagagliflozin, empagliflozin 25 mg had higher quality and lower cost, making it an absolute advantage choice. In other cases of the OWSA, compared with dapagliflozin, empagliflozin 25 mg was associated with increased QALYs and cost, while the ICUR was below the threshold. The changes in key parameters altered the results of the economic analysis, indicating that the cost-effectiveness advantage of treatment regimen of empagliflozin 25 mg was not robust (Supplementary Table S5).

**Figure 4:**
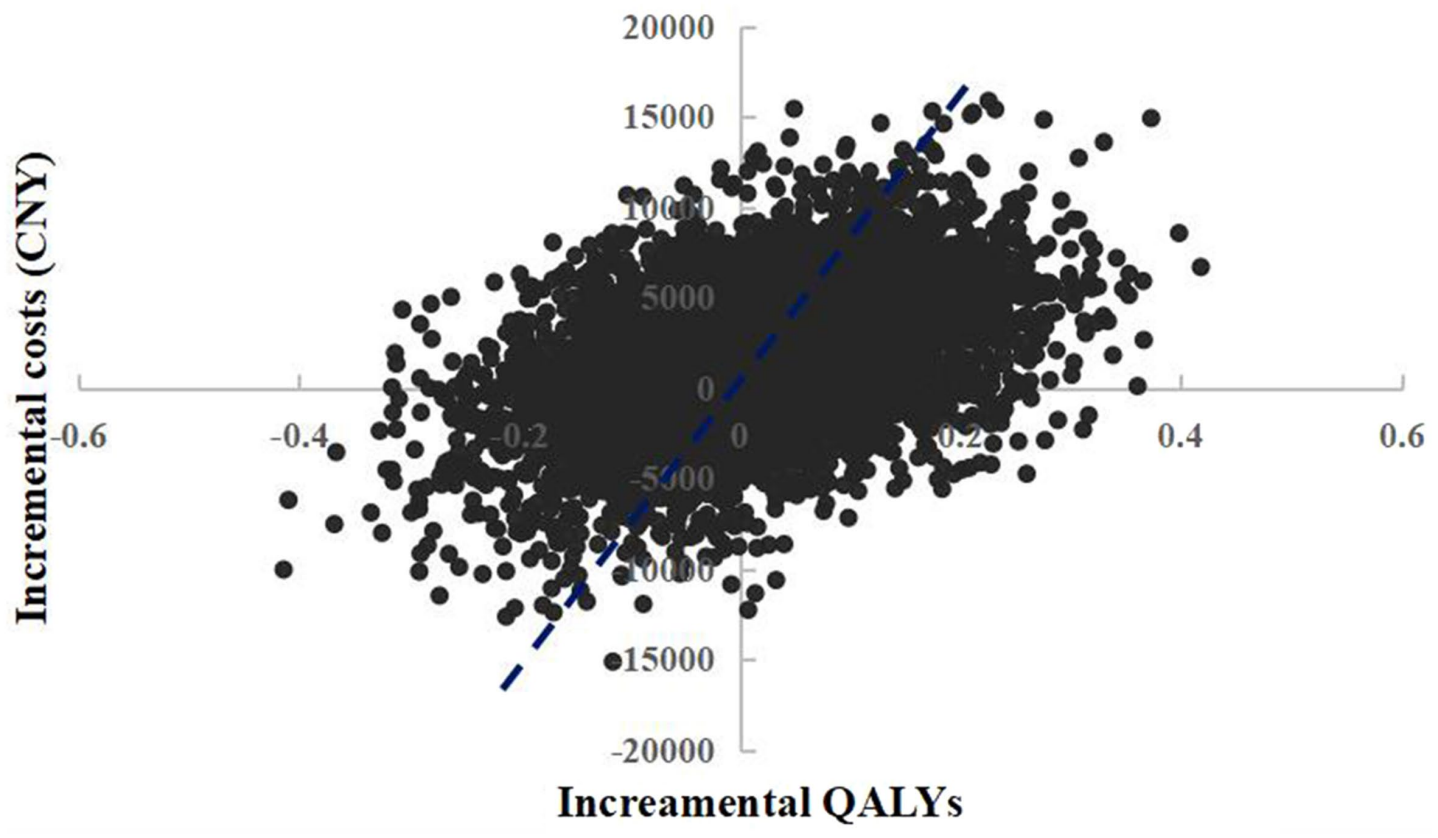
Probabilistic sensitivity analysis scatterplot (empagliflozin 25 mg vs. dapagliflozin 10 mg).

The scatter plot of change in cost and change in QALYs in PSA was shown in Figure 4. With a WTP threshold of CNY 85,698/QALY, there was a 47.2% probability that empagliflozin 25 mg was more cost-effective than dapagliflozin 10 mg.

However, in short-term scenario analysis, the treatment group supplemented with empagliflozin 25 mg showed higher QALYs and costs compared to dapagliflozin 10 mg, and the ICUR was higher than the threshold (Table 6). This result could not further support the cost-effectiveness advantage of the empagliflozin 25 mg treatment regimen observed in the base-case analysis.

**Table 6:** Breakdown of costs for the shorter-time scenario analyses.

Cost category (CNY)	Metformin + empagliflozin 25 mg	Metformin + dapagliflozin 10 mg	Difference
Total cost	33,552	25,665	7,887
Treatment	17,248	9,260	7,988
Management	6,030	6,034	−3
CVD	4,498	4,525	−27
Renal	677	673	4
Ulcer/amputation/neuropathy	4,012	4,072	−60
Eye	1,071	1,072	−1
NSHE	14	29	−14
SHE1	–	–	–
SHE2	–	–	–

NSHE, non-severe hypoglycemic event; SHE1, severe hypoglycemic event (not requiring medical assistance); SHE2, severe hypoglycemic event (requiring medical assistance).

## 4 Discussion

T2DM is a long-term chronic metabolic disease, which not only seriously endangers the health of patients, but also brings huge economic burden to patients and their families. Therefore, when selecting the optimal treatment scheme for T2DM patients, it is important to consider not only the short-term efficacy and safety, but also the long-term health outputs and health costs.

In this study, the people concerned were T2DM patients who were poorly controlled with the first-line metformin monotherapy in China. The CORE model was used to simulate the long-term disease progression and health outcomes of patients treated with empagliflozin and dapagliflozin. According to the drug instructions and diabetes treatment guidelines, the initial recommended dose of dapagliflozin is 5 mg for T2DM patients with normal liver and kidney functions. In patients who tolerate this product and require further blood sugar control, as well as in patients with heart failure and chronic kidney disease, the dose could be increased to 10 mg. According to the drug manual of empagliflozin and the treatment guidelines, the recommended dose of empagliflozin was 10 mg qd for T2DM patients with normal liver and kidney functions, which could be increased to 25 mg for patients who tolerate this product and require further blood sugar control. At present, the original drugs of dapagliflozin and empagliflozin are both marketed in China with a specification of 10 mg. Therefore, this study conducted a cost-utility analysis of dapagliflozin 10 mg vs. empagliflozin 10 mg. In addition, a cost-utility analysis of dagliagazine 10 mg vs. Engliagazine 25 mg was also performed as a reference. The HbA1c data used in this research were derived from a published network meta-analysis, which simultaneously covered the efficacy data of dapagliflozin 10 mg, empagliflozin 10 mg and empagliflozin 25 mg from multiple centers, ensuring the consistency and comparability of efficacy parameters among the studied groups (31). Regarding cardiovascular and renal outcomes, dapagliflozin and different doses of empagliflozin might lead to differences (11, 35). However, the IQVIA CORE diabetes model primarily based on glycemic efficacy during the process of conducting cost-utility analysis.

Based on the published results and data from our modeling analysis, we have determined that both the treatment regimen of empagliflozin 10 mg and empagliflozin 25 mg could result in longer survival time and quality adjusted life years in patients compared to dapagliflozin 10 mg, thanks to the superior hypoglycemic effect and cardiovascular protective effect for empagliflozin (36, 37).

Among them, the treatment group of the combination for metformin combined with empagliflozin 10 mg generated lower direct medical expenses than the combination of metformin and dapagliflozin 10 mg, which was an absolutely economical choice from a long-term perspective. In order to verify the robustness of the results, a series of one-way sensitivity analysis were carried out. The time frame of the simulation study was adjusted based on the differences in patient lifespan. Different discount rates were used according to the guidelines, and the medication costs of the oral hypoglycemic drugs studied were adjusted according to the differences in the actual bid prices in centralized drug procurement. The OWSA results further supported the robustness of the results, demonstrating that empagliflozin 10 mg was a more economical treatment option than dapagliflozin 10 mg. In this economic study, the direct medical costs included in the short-term and long-term simulation time could be different, and the long-term simulation took more account of the cost for treating complications. Because of the different emphasis of different time periods, it was more comprehensive to combine the evaluations for two time frames. We have shortened the time frame of the simulation study to 4 years and conducted short-term scenario analysis, and the cost-utility advantage of the empagliflozin 10 mg treatment regimen was still evident. Due to the fact that the original drugs of dapagliflozin and empagliflozin marketed in China were both with a specification of 10 mg, above results had important guiding significance for the selection of treatment drugs for T2DM patients poorly controlled with metformin monotherapy in China.

It was found that the direct medical expenses incurred by the combined treatment group of metformin with empagliflozin 25 mg were slightly higher than those of the treatment group of metformin with dapagliflozin 10 mg. However, the ICUR was lower than the set WTP threshold, confirming the economic advantage of empagliflozin 25 mg. Similarly, the one-way sensitivity analysis was conducted to validate the robustness of the results. The changes in key parameters altered the results of the economic analysis, indicating that the cost-utility advantage of the treatment scheme for empagliflozin 25 mg was not robust. However, it was worth noting that when the price of empagliflozin adopted the winning bid price in centralized procurement, empagliflozin 25 mg showed better efficacy and lower cost compared to dapagliflozin, making it an absolute advantage choice. This fully verified the important role of China’s centralized drug procurement policy in reducing the treatment burden on patients.

However, in the probabilistic sensitivity analysis of the economic evaluation of different specifications of empagliflozin and dapagliflozin 10 mg, the probability of empagliflozin being more cost-effective than dapagliflozin was not significant. This was because that diabetes was a long-term chronic disease, and the incremental QALYs brought by hypoglycemic drugs were very limited, which were consistent with the published literatures. A recent long-term cost-effectiveness study suggested that dapagliflozin plus standard treatment was expected to generate an additional 0.25 QALYs compared to standard treatment (38). Another cost-effectiveness analysis showed that the QALYs increment of iGlarLixi (glargine insulin 100 U/mL plus lixisenatide) and iDegLira (degludec insulin plus liraglutide) were only 0.015 (39). The two drugs evaluated in this study (dapagliflozin and empagliflozin) were both belong to SGLT-2i with similar mechanisms of action, and their differences in efficacy and adverse reactions were small. Therefore, the empagliflozin treatment regimen did not show significant advantages in the probabilistic sensitivity analysis.

This study had the following advantages. Many of the latest data, including drug prices and utility values, were used to ensure that this economic evaluation was based on the latest evidence and met the requirements of continuously updating in health economic evaluation, supporting the medical decisions. Numerous sensitivity analyses were performed and the results were found to be robust for changes in key parameters. Taking into account the drug specifications listed in China, the commonly used clinical specifications for dapagliflozin and empagliflozin were both 10 mg. Studies have shown that for T2DM patients with poor glycemic control after the monotherapy with metformin, the cost-utility of the second-line treatment combining metformin with empagliflozin 10 mg had an absolute advantage over the combination of dapagliflozin 10 mg and metformin. Under the background that empagliflozin being involved in the centralized procurement in China, the economic advantages of empagliflozin would be more prominent.

But it should be aware that there are some limitations in our analysis. Firstly, this study was carried out based on limited data. The baseline demographic and biochemical parameters of the model cohort were mainly derived from the EMPA-REG MET trial, which could not fully represent the real Chinese T2DM patients. Given the lack of direct comparison data between empagliflozin and dapagliflozin in randomized controlled trials, the therapeutic effects applied in the analysis were based on network meta-analysis. If there are high-quality head-to-head study data, giving priority to using Chinese local randomized controlled trial or real-world study data would make the research conclusions more convincing. In addition, to enhance the operability and stability of the model during long-term simulation process, this study simplified the next step treatment plan after the combined treatment of metformin and SGLT2 inhibitors with poor hypoglycemic effects, which would be more complicated in clinical practice. Furthermore, this study assessed cost-utility analysis mainly based on glycemic efficacy, without incorporating cardio-renal outcome data, which was most applicable to patients whose primary therapeutic goal was glycemic control. It is suggested that future economic studies should incorporate multi-dimensional clinical endpoints based on the high-quality real-world data of Chinese local patients to enhance the clinical applicability and promotional value of research findings. Therefore, caution should be exercised in interpreting and utilizing these research findings.

## 5 Conclusion

For T2DM patients with insufficient control by metformin monotherapy, a 40-year simulation suggested that the addition of empagliflozin 10 mg was an absolutely economical treatment option with better efficacy and lower cost compared to dapagliflozin 10 mg. Sensitivity analysis confirmed the robustness of the research results. Based on the set WTP threshold, empagliflozin 25 mg was more cost-effective than dapagliflozin 10 mg. Sensitivity analysis confirmed the important role of China’s centralized drug procurement policy in reducing the treatment burden on patients. These results of this study provide an important reference for Chinese patients with type 2 diabetes to make effective use of medical resources.

## Data Availability

The original contributions presented in the study are included in the article/Supplementary material, further inquiries can be directed to the corresponding authors.
