# Supplementary material for: Cost-utility analysis of empagliflozin compared with dapagliflozin in patients with type 2 diabetes in China
Source: Front Public Health. 2025 Jul 7;13:1566101. doi: 10.3389/fpubh.2025.1566101 (PMC12277269; doi:10.3389/fpubh.2025.1566101)
Supplement: Supplementary file 1 [file Data_Sheet_1.docx]

Supplementary Material

Supplementary Tables

**Supplementary Table S1** Baseline characteristics of the patients in the trial

| **Variable** | **Mean ± SE** | | | **Sources** |
| --- | --- | --- | --- | --- |
| **Patient demographics** | | |  |  |
| Start age (years) | | 55.7 ± 9.9 | | EMPA-REG MET |
| Duration of diabetes (years) | | 6 | | (1) |
| Proportion male (0-1) | | 0.57 | | EMPA-REG MET |
| **Baseline risk factors** | | | | |
| HbA1c (%-points) | | 7.9 ± 0.85 | | EMPA-REG MET |
| SBP (mmHg) | | 129.4 ± 14.6 | | EMPA-REG MET |
| DBP (mmHg) | | 78.7 ± 8.1 | | EMPA-REG MET |
| T-CHOL (mg/dL) | | 81.00 ± 0.066 | | EMPA-REG MET |
| HDL (mg/dL) | | 22.68 ± 0.02 | | EMPA-REG MET |
| LDL (mg/dL) | | 44.10 ± 0.06 | | EMPA-REG MET |
| TRIG (mg/dL) | | 34.56 ± 0.088 | | EMPA-REG MET |
| BMI (kg/m^2^) | | 29.2 ± 5.5 | | EMPA-REG MET |
| eGFR (mL/min/1.73m^2^) | | 89.0 ± 20.1 | | EMPA-REG MET |
| Haemoglobin (mg/dL) | | 13.85 ± 1.35 | | (1) |
| WBC (10^6^/mL) | | 6.9 ± 1.45 | | (1) |
| Heart rate (bpm) | | 72 | | default |
| WHR | | 0.9 | | default |
| uACR (mg/mmoL) | | 4.5 ± 15.17 | | (1) |
| Serum creatinine (mg/dL) | | 0.88 ± 0.12 | | (1) |
| Serum albumin (g/dL) | | 4.75 ± 0.41 | | (1) |
| Prop. smoker | | 0.273 | | Chinese Center for Disease Control and Prevention |
| Cigarettes/day | | 5 | | default |
| Alcohol consumption oz/week | | 5 | | Global Status Report on Alcohol and health 2018 |
| **Racial characteristics** | | | | |
| Prop. White | | 0 | | default |
| Prop. Black | | 0 | | default |
| Prop. Hispanic | | 0 | | default |
| Prop. Native American | | 0 | | default |
| Prop. Asian/Pacific Islander | | 1 | | default |
| Prop. Australian (South Europ) | | 0 | | default |
| Prop. Australian (Aboriginal) | | 0 | | default |
| **Baseline CVD complications** | | | | |
| Prop. MI | | 0.013 | | (1) |
| Prop. angina | | 0.005 | | (1) |
| Prop. PVD | | 0.001 | | (1) |
| Prop. stroke | | 0.060 | | (1) |
| Prop. HF | | 0.002 | | (1) |
| Prop. atrial fibrillation | | 0.013 | | (1) |
| Prop. LVH | | 0.006 | | (1) |
| **Baseline renal complications** | | | | |
| Prop. microalbuminuria | | 0.027 | | (1) |
| Prop. gross proteinuria | | 0.04 | | (2) |
| Prop. ESRD | | 0.00118 | | (1) |
| **Baseline retinopathy complications** | | | | |
| Prop. BDR | 0.047 | | | (1) |
| Prop. PDR | 0.071 | | | (1) |
| Prop. SVL | 0.030 | | | (1) |
| Prop. ME | 0.001 | | | (1) |
| Prop. cataract | 0.058 | | | (1) |
| **Baseline foot ulcer complications** | | | | |
| Prop. uninfected ulcer | 0.0043 | | | (2) |
| Prop. infected ulcer | 0.0043 | | | (2) |
| Prop. healed ulcer | 0 | | | (2) |
| Prop. history of amputation | 0.0043 | | | (2) |
| **Baseline neuropathy** | | | | |
| Prop. neuropathy | 0.082 | | | (1) |
| **Baseline depression** | | | | |
| Prop. depression | 0 | | | No source |

SE: standard error; SBP: systolic blood pressure; DBP: diastolic blood pressure; T-Chol: total cholesterol; HDL: high density lipoprotein; LDL: low density lipoprotein; TRIG: triglycerides; BMI: body mass index; WBC: white blood cells; uACR: urinary albumin/creatinine ratio; MI: myocardial infarction; PVD: peripheral vascular disease; HF: heart failure; LVH: left ventricular hypertrophy; ESRD: end-stage renal disease; BDR: background diabetic retinopathy; PDR: proliferative diabetic retinopathy; SVL: severe vision loss; ME: macular edema.

**Supplementary Table S2** Clinical input parameters applied in the cost-utility analysis

|  | **Metformin+** **Empagliflozin 10 mg** | **Metformin+** **Empagliflozin 25 mg** | **Metformin+** **Dapagliflozin 10 mg** | **Metformin+ Insulin glargine** |
| --- | --- | --- | --- | --- |
| Change from baseline HbA1c (%) | -0.7 | -0.77 | -0.67 | -0.74 |
| SHE rate (per 100 patient-years) | 0 | 0 | 0 | 1 |
| NSHE rate (per 100 patient-years) | 1.8 | 1.4 | 4 | 32 |
| UTI rate (per 100 patient-years) | 5.1 | 5.6 | 7 | _ |

NSHE: nonsevere hypoglycemia; SHE: severe hypoglycemia；UTI：urinary tract infection.

**Supplementary Table S3** Utility data of health state and disutility associated to events

| Variable | Mean ± SD | Reference |
| --- | --- | --- |
| U T2 no complications | 0.876 ± 0.053 | (1) |
| U T1 no complications | 0.814 ± 0.000 | default |
| DisU MI event | -0.055 ± 0.007 | (1) |
| U post MI | 0.821 ± 0.007 | (1) |
| U angina | 0.786 ± 0.018 | (1) |
| U heart failure | 0.768 ± 0.030 | (1) |
| DisU stroke event | -0.164 ± 0.030 | (1) |
| U post stroke | 0.712 ± 0.030 | (1) |
| U PVD | 0.815 ± 0.022 | (1) |
| U microalbuminuria | 0.876 ± 0.053 | (1) |
| U macroalbuminuria | 0.828 ± 0.022 | (1) |
| U HD | 0.712 ± 0.056 | (1) |
| U PD | 0.672 ± 0.138 | (1) |
| U RT | 0.762 ± 0.053 | (1) |
| U BDR | 0.836 ± 0.062 | (1) |
| U BDR wrongly treated | 0.836 ± 0.062 | (1) |
| U PDR laser treated | 0.806 ± 0.015 | (1) |
| U PDR no laser | 0.806 ± 0.015 | (1) |
| U ME | 0.836 ± 0.013 | (1) |
| U SVL | 0.802 ± 0.025 | (1) |
| U cataract | 0.860 ± 0.008 | (1) |
| U neuropathy | 0.792 ± 0.014 | (1) |
| U healed ulcer | 0.876 ± 0.053 | (1) |
| U active ulcer | 0.706 ± 0.561 | (1) |
| DisU AMP event | -0.28 ± 0.011 | default |
| U post amputation | 0.596 ± 0.056 | (1) |
| DisU for NSHE (during daytime) | -0.005 ± 0.0015 | default |
| DisU for NSHE (nocturnal) | -0.007 ± 0.025 | default |
| DisU for SHE 1 (during daytime) | -0.021 | (1) |
| DisU for SHE 1 (nocturnal) | -0.021 | (1) |
| DisU for SHE 2 (during daytime) | -0.074 | (1) |
| DisU for SHE 2 (nocturnal) | -0.074 | (1) |
| DisU for UTI/GI | -0.005 | (1) |
| DisU keto event | 0 ± 0 | default |
| DisU edema event | -0.01 ± 0.00 | default |
| U post edema | 0.785 ± 0.007 | default |
| U depression not treated | 0.785 ± 0.000 | default |
| U depression treated | 0.785 ± 0.000 | default |

DisU: disutility of an event; U: health state utility; T2: type 2 diabetes mellitus; T1: type 1 diabetes mellitus; AMP: amputation; BDR: background retinopathy; PDR: proliferative diabetic retinopathy; HD: hemodialisis; PD: peritoneal dialysis; ME: macular edema; MI: myocardial infarction; PVD: peripheral vascular disease; RT: renal transplant; SVL: severe vision loss; UTI: urinary tract infection; GI: genital infection.

**Supplementary Table S4** Cost-utility results of empagliflozin 10 mg vs. dapagliflozin 10 mg

|  | **Dapagliflozin** | **Empagliflozin 10 mg** | **Incremental** | |  |
| --- | --- | --- | --- | --- | --- |
| **Base case** | | | |  |  |
| Life expectancy (years) | 12.493 | 12.504 | 0.011 | |  |
| Quality-adjusted life expectancy (years) | 8.963 | 8.974 | 0.011 | |  |
| Direct costs (CNY) | 171,561 | 171,282 | -279 | |  |
| ICUR |  |  | -25,364 | |  |
| **Empagliflozin adopted the lowest winning bid prices in the centralized medicine procurement** | | | | | |
| Life expectancy (years) | 12.493 | 12.504 | 0.011 | |  |
| Quality-adjusted life expectancy (years) | 8.963 | 8.974 | 0.011 | |  |
| Direct costs (CNY) | 171,561 | 170,418 | -1,142 | |  |
| ICUR |  |  | -103,818 | |  |
| **Empagliflozin adopted the highest winning bid prices in the centralized medicine procurement** | | | |  |  |
| Life expectancy (years) | 12.493 | 12.504 | 0.011 | |  |
| Quality-adjusted life expectancy (years) | 8.963 | 8.974 | 0.011 | |  |
| Direct costs (CNY) | 171,561 | 170,485 | -1,075 | |  |
| ICUR |  |  | -97,727 | |  |
| **Metformin adopted the lowest winning bid prices in the centralized medicine procurement** | | | |  |  |
| Life expectancy (years) | 12.493 | 12.504 | 0.011 | |  |
| Quality-adjusted life expectancy (years) | 8.963 | 8.974 | 0.011 | |  |
| Direct costs (CNY) | 158,841 | 158,552 | -289 | |  |
| ICUR |  |  | -26,273 | |  |
| **Metformin adopted the highest winning bid prices in the centralized medicine procurement** | | | |  |  |
| Life expectancy (years) | 12.493 | 12.504 | 0.011 | |  |
| Quality-adjusted life expectancy (years) | 8.963 | 8.974 | 0.011 | |  |
| Direct costs (CNY) | 159,146 | 158,857 | -289 | |  |
| ICUR |  |  | -26,273 | |  |
| **Dapagliflozin adopted the lowest winning bid prices in consistency evaluation** | | | |  |  |
| Life expectancy (years) | 12.493 | 12.504 | 0.011 | |  |
| Quality-adjusted life expectancy (years) | 8.963 | 8.974 | 0.011 | |  |
| Direct costs (CNY) | 171,515 | 171,282 | -234 | |  |
| ICUR |  |  | -21,273 | |  |
| **Dapagliflozin adopted the highest winning bid prices in consistency evaluation** | | | |  |  |
| Life expectancy (years) | 12.493 | 12.504 | 0.011 | |  |
| Quality-adjusted life expectancy (years) | 8.963 | 8.974 | 0.011 | |  |
| Direct costs (CNY) | 171,595 | 171,282 | -314 | |  |
| ICUR |  |  | -28,545 | |  |
| **Discount rate = 0%** | | | |  |  |
| Life expectancy (years) | 22.57 | 22.589 | 0.011 | |  |
| Quality-adjusted life expectancy (years) | 15.93 | 15.949 | 0.019 | |  |
| Direct costs (CNY) | 352,268 | 351,849 | -419 | |  |
| ICUR |  |  | -22,053 | |  |
| **Discount rate = 3%** | | | |  |  |
| Life expectancy (years) | 15.45 | 15.464 | 0.013 | |  |
| Quality-adjusted life expectancy (years) | 11.021 | 11.035 | 0.014 | |  |
| Direct costs (CNY) | 222,680 | 222,364 | -316 | |  |
| ICUR |  |  | -22,571 | |  |
| **Discount rate = 8%** | | | |  |  |
| Life expectancy (years) | 9.53 | 9.537 | 0.008 | |  |
| Quality-adjusted life expectancy (years) | 6.885 | 6.893 | 0.008 | |  |
| Direct costs (CNY) | 122,742 | 122,499 | -242 | |  |
| ICUR |  |  | -30,250 | |  |
| **Time horizon = 10 years** | | | |  |  |
| Life expectancy (years) | 7.333 | 7.332 | -0.001 | |  |
| Quality-adjusted life expectancy (years) | 5.402 | 5.403 | 0.001 | |  |
| Direct costs (CNY) | 79,031 | 78,798 | -232 | |  |
| ICUR |  |  | -232,000 | |  |
| **Time horizon = 20 years** | | | |  |  |
| Life expectancy (years) | 10.962 | 10.967 | 0.005 | |  |
| Quality-adjusted life expectancy (years) | 7.936 | 7.942 | 0.006 | |  |
| Direct costs (CNY) | 138,435 | 138,207 | -228 | |  |
| ICUR |  |  | -38,000 | |  |
| **Time horizon = 30 years** | | | |  |  |
| Life expectancy (years) | 12.245 | 12.258 | 0.013 | |  |
| Quality-adjusted life expectancy (years) | 8.799 | 8.809 | 0.01 | |  |
| Direct costs (CNY) | 165,402 | 165,595 | 193 | |  |
| ICUR |  |  | 19,310 | |  |
| **Time horizon = 50 years** | | | |  |  |
| Life expectancy (years) | 12.509 | 12.517 | 0.008 | |  |
| Quality-adjusted life expectancy (years) | 8.972 | 8.980 | 0.008 | |  |
| Direct costs (CNY) | 171,973 | 171,772 | -201 | |  |
| ICUR |  |  | -25,125 | |  |

**Supplementary Table S5** Cost-utility results of empagliflozin 25 mg vs. dapagliflozin 10 mg

|  | **Dapagliflozin** | **Empagliflozin 25 mg** | **Incremental** |  |
| --- | --- | --- | --- | --- |
| **Base case** | | | | |
| Life expectancy (years) | 12.493 | 12.513 | 0.02 |  |
| Quality-adjusted life expectancy (years) | 8.963 | 8.983 | 0.02 |  |
| Direct costs (CNY) | 171,561 | 173,162 | 1,601 |  |
| ICUR |  |  | 80,052 |  |
| **Empagliflozin adopted the lowest winning bid prices in the centralized medicine procurement** | | | | |
| Life expectancy (years) | 12.493 | 12.513 | 0.02 |  |
| Quality-adjusted life expectancy (years) | 8.963 | 8.983 | 0.02 |  |
| Direct costs (CNY) | 171,561 | 170,709 | -851 |  |
| ICUR |  |  | -42,550 |  |
| **Empagliflozin adopted the highest winning bid prices in the centralized medicine procurement** | | | | |
| Life expectancy (years) | 12.493 | 12.513 | 0.02 |  |
| Quality-adjusted life expectancy (years) | 8.963 | 8.983 | 0.02 |  |
| Direct costs (CNY) | 171,561 | 170,775 | -785 |  |
| ICUR |  |  | -39,350 |  |
| **Metformin adopted the lowest winning bid prices in the centralized medicine procurement** | | | | |
| Life expectancy (years) | 12.493 | 12.513 | 0.02 |  |
| Quality-adjusted life expectancy (years) | 8.963 | 8.983 | 0.02 |  |
| Direct costs (CNY) | 158,841 | 160,423 | 1,582 |  |
| ICUR |  |  | 79,116 |  |
| **Metformin adopted the highest winning bid prices in the centralized medicine procurement** | | | | |
| Life expectancy (years) | 12.493 | 12.513 | 0.02 |  |
| Quality-adjusted life expectancy (years) | 8.963 | 8.983 | 0.02 |  |
| Direct costs (CNY) | 159,146 | 160,729 | 1,583 |  |
| ICUR |  |  | 79,138 |  |
| **Dapagliflozin adopted the lowest winning bid prices in consistency evaluation** | | | | |
| Life expectancy (years) | 12.493 | 12.504 | 0.02 |  |
| Quality-adjusted life expectancy (years) | 8.963 | 8.983 | 0.02 |  |
| Direct costs (CNY) | 171,515 | 173,162 | 1,646 |  |
| ICUR |  |  | 82,313 |  |
| **Dapagliflozin adopted the highest winning bid prices in consistency evaluation** | | | | |
| Life expectancy (years) | 12.493 | 12.513 | 0.02 |  |
| Quality-adjusted life expectancy (years) | 8.963 | 8.983 | 0.02 |  |
| Direct costs (CNY) | 171,595 | 173,162 | 1,566 |  |
| ICUR |  |  | 78,312 |  |
| **Discount rate = 0%** | | | | |
| Life expectancy (years) | 22.57 | 22.621 | 0.02 |  |
| Quality-adjusted life expectancy (years) | 15.93 | 15.976 | 0.046 |  |
| Direct costs (CNY) | 352,268 | 353,648 | 1,380 |  |
| ICUR |  |  | 30,006 |  |
| **Discount rate = 3%** | | | | |
| Life expectancy (years) | 15.45 | 15.479 | 0.028 |  |
| Quality-adjusted life expectancy (years) | 11.021 | 11.05 | 0.027 |  |
| Direct costs (CNY) | 222,680 | 224,217 | 1,537 |  |
| ICUR |  |  | 56,927 |  |
| **Discount rate = 8%** | | | | |
| Life expectancy (years) | 9.53 | 9.542 | 0.012 |  |
| Quality-adjusted life expectancy (years) | 6.885 | 6.897 | 0.013 |  |
| Direct costs (CNY) | 122,742 | 124,399 | 1,657 |  |
| ICUR |  |  | 127,491 |  |
| **Time horizon = 10 years** | | | | |
| Life expectancy (years) | 7.333 | 7.335 | 0.002 |  |
| Quality-adjusted life expectancy (years) | 5.402 | 5.407 | 0.005 |  |
| Direct costs (CNY) | 79,031 | 80,813 | 1,783 |  |
| ICUR |  |  | 356,508 |  |
| **Time horizon = 20 years** | | | | |
| Life expectancy (years) | 10.962 | 10.973 | 0.012 |  |
| Quality-adjusted life expectancy (years) | 7.936 | 7.949 | 0.013 |  |
| Direct costs (CNY) | 138,435 | 140,288 | 1,853 |  |
| ICUR |  |  | 142,521 |  |
| **Time horizon = 30 years** | | | | |
| Life expectancy (years) | 12.245 | 12.254 | 0.009 |  |
| Quality-adjusted life expectancy (years) | 8.799 | 8.810 | 0.01 |  |
| Direct costs (CNY) | 165,402 | 167,160 | 1,758 |  |
| ICUR |  |  | 175,762 |  |
| **Time horizon = 50 years** | | | | |
| Life expectancy (years) | 12.509 | 12.533 | 0.025 |  |
| Quality-adjusted life expectancy (years) | 8.972 | 8.996 | 0.024 |  |
| Direct costs (CNY) | 171,973 | 173,796 | 1,822 |  |
| ICUR |  |  | 75,934 |  |

**References**

1. Salem A, Men P, Ramos M, Zhang YJ, Ustyugova A, Lamotte M. Cost-effectiveness analysis of empagliflozin compared with glimepiride in patients with type 2 diabetes in China. *J Comp Eff Res.* (2021) 10:469-480. doi: 10.2217/cer-2020-0284

2. Wu J，He X，Liu Y. Cost-effectiveness analysis of insulin aspart 30 versus insulin glargine in patients with type 2 diabetes in China. *Chin Pharm J.* (2016) 51:242-247. doi: 10. 11669/cpj. 2016. 03. 021
